# Targeting macrophages and ion homeostasis in T2D: new genes and therapeutic pathways identified

**DOI:** 10.3389/fimmu.2025.1514243

**Published:** 2025-08-14

**Authors:** Lisha Mou, Tony Bowei Wang, Ying Lu, Zijing Wu, Yuxian Chen, Ziqi Luo, Xinyu Wang, Zuhui Pu

**Affiliations:** ^1^ Institute of Translational Medicine, Health Science Center, The First Affiliated Hospital of Shenzhen University, Shenzhen Second People’s Hospital, Shenzhen, Guangdong, China; ^2^ MetaLife Center, Shenzhen Institute of Translational Medicine, Shenzhen, Guangdong, China; ^3^ Biology Department, Skidmore College, Saratoga Springs, NY, United States; ^4^ Department of Endocrinology, The First Affiliated Hospital of Shenzhen University, Shenzhen Second People’s Hospital, Shenzhen, Guangdong, China; ^5^ Imaging Department, The First Affiliated Hospital of Shenzhen University, Shenzhen Second People’s Hospital, Shenzhen, Guangdong, China

**Keywords:** type 2 diabetes (T2D), macrophages, ion homeostasis, immune infiltration, ScRNA-seq, machine learning, cytokine signatures, therapeutic targets

## Abstract

**Introduction:**

Type 2 diabetes (T2D) is characterized by insulin resistance and chronic inflammation, with macrophages playing a crucial role in pancreatic islet dysfunction. This study explored the intersection of macrophage-specific gene expression and abnormal blood monovalent inorganic cation concentration-related genes (ABRGs) in T2D patients via single-cell RNA sequencing (scRNA-seq) and machine learning to identify key genes and potential therapeutic targets.

**Methods:**

ScRNA-seq data from the pancreatic islet cells of 27 nondiabetic (ND) patients and 17 T2D patients were analyzed to identify differentially expressed genes (DEGs) in macrophages. These DEGs were intersected with ABRGs to identify hub genes. Machine learning models were developed to predict T2D, and structural predictions of the hub proteins were performed. PPI networks and regulatory networks involving transcription factors (TFs) and miRNAs were also analyzed. Correlations between hub ABRGs and immune cell infiltration, as well as cytokine responses, were examined via ssGSEA and immune response enrichment analysis (IREA).

**Results:**

Sixteen overlapping hub ABRGs, including *ATP1A1*, *CACNA1D*, and *CLDN10*, were identified. The GBM model demonstrated high predictive accuracy, with an AUC of 0.988. Correlation analysis revealed significant relationships between the hub genes and the infiltration of immune cells, particularly macrophages. Cytokine enrichment analysis revealed that macrophages in T2D exhibit a distinct signature of cytokines, including IL15, IFNα1, IFNβ, and IL17F. PPI networks highlighted significant interactions among the hub genes. Regulatory network analysis revealed that STAT3 is a central TF and that miRNAs such as hsa-mir-1-3p are critical regulators.

**Discussion:**

This study highlights the central roles of macrophages and ABRGs in T2D, identifying novel genes and regulatory networks that contribute to disease progression. The integration of scRNA-seq and machine learning provides valuable insights and potential therapeutic targets for T2D.

## Introduction

Type 2 diabetes (T2D) is a multifaceted metabolic disorder characterized by insulin resistance, chronic hyperglycemia, and systemic inflammation (1). Among the various immune cells that infiltrate pancreatic islet tissues, macrophages have emerged as key players in the pathogenesis of T2D (2). Macrophages are highly plastic immune cells that can adopt proinflammatory (M1) or anti-inflammatory (M2) phenotypes in response to microenvironmental signals (3). In T2D, there is a notable shift toward the proinflammatory M1 phenotype, which contributes to the chronic inflammation observed in the pancreatic islets (4). This inflammation exacerbates insulin resistance and impairs β-cell function, ultimately leading to the progressive loss of insulin secretion capacity (5). The role of macrophages in T2D is thus critical, as they not only participate in the immune response but also directly influence the metabolic dysregulation characteristic of the disease (6).

In recent years, interest in the role of inorganic ions, particularly monovalent cations such as sodium (Na+) and potassium (K+), in the development and progression of T2D, has increased (7). To investigate this, our study focuses on “Abnormal blood monovalent inorganic cation concentration-related genes” (ABRGs). This term, as used herein, refers to a specific gene set titled “HP_ABNORMAL_BLOOD_MONOVALENT_INORGANIC_CATION_CONCENTRATION,” sourced directly from the Molecular Signatures Database (MSigDB) (8, 9). MSigDB is a well-established and widely utilized public resource containing curated gene sets that represent various biological states and processes. The ABRG set we employed encompasses genes associated with abnormal concentrations of these critical monovalent inorganic cations. These ABRGs are involved in various physiological processes pertinent to T2D, such as insulin secretion, cellular metabolism, and inflammatory signaling pathways, all of which can be influenced by the concentration and balance of monovalent cations like Na+ and K+. For example, disturbances in potassium levels have been linked to impaired insulin secretion and β-cell dysfunction, whereas sodium imbalance can influence blood pressure and vascular function, both of which are often dysregulated in T2D patients (10, 11). The identification of genes associated with ABRGs has provided new insights into how ion homeostasis may contribute to the metabolic and inflammatory processes underlying T2D.

Advancements in single-cell RNA sequencing (scRNA-seq) technology have revolutionized the study of complex diseases such as T2D by enabling high-resolution profiling of gene expression at the level of individual cells (12). This technique allows for the dissection of cellular heterogeneity within tissues, making it possible to identify distinct cell populations and their specific contributions to disease pathogenesis (13). In the context of T2D, scRNA-seq has been instrumental in revealing the diverse roles of various cell types within pancreatic islets, including β-cells, α-cells, and immune cells such as macrophages (14, 15). By characterizing the transcriptomic landscape of these cells, researchers can gain deeper insights into the cellular and molecular mechanisms driving T2D and identify potential therapeutic targets.

Machine learning, another rapidly evolving field, has become an invaluable tool in the analysis of complex biological data (16, 17). In T2D research, machine learning algorithms are increasingly used to develop predictive models that can identify patients at risk of developing the disease, stratify patients on the basis of disease severity, and predict responses to treatment (18). These algorithms can analyze large datasets, such as those generated by scRNA-seq, to uncover patterns and relationships that might not be apparent through traditional statistical methods (19, 20). By integrating machine learning with scRNA-seq data, researchers can create robust models that not only predict disease outcomes but also enhance our understanding of the underlying biological processes.

Given the central roles of macrophages and ABRGs in T2D pathophysiology, this study aims to explore the intersection of these two critical factors by cutting-edge technologies. We used scRNA-seq to identify differentially expressed genes (DEGs) in macrophages from T2D and nondiabetic pancreatic islet tissues. We then intersected these macrophage-specific DEGs with ABRG-related genes to pinpoint hub genes that may play pivotal roles in T2D. These hub genes were subsequently used to develop predictive models for T2D via machine learning techniques, and their potential functions were further explored through structural predictions and network analyses.

This comprehensive approach, which integrates macrophage biology, ion homeostasis, single-cell sequencing, and machine learning, provides a deeper understanding of the molecular mechanisms driving T2D. This study also highlights potential therapeutic targets that could modulate both immune and metabolic pathways in this disease.

## Methods

### Single-cell RNA sequencing and gene expression profiling

To explore the gene expression profiles of pancreatic islet cells, we obtained single-cell RNA sequencing (scRNA-seq) data from 27 nondiabetic (ND) individuals and 17 type 2 diabetes (T2D) patients (21). The data were processed and analyzed via standard scRNA-seq workflows via Seurat (v4.4.0), including quality control, normalization, and clustering. To maintain data quality, we applied the following filtering criteria: cells with a mitochondrial content less than 15%, more than 500 cells, and a gene count ranging from 1,000-25,000 were retained. The UMAP method was employed to visualize the clustering of cells into distinct cell types within the islets. Seven primary cell types were identified, including endocrine cells, stellate cells, endothelial cells, mast cells, ductal cells, acinar cells, and macrophages. The established marker genes were used to annotate the cell clusters (21), and the proportions of these cell types were compared between the T2D and ND samples. Additionally, individual-level analyses were conducted to assess the heterogeneity of cellular compositions across samples.

### Gene set variation analysis

For GSVA, hallmark gene sets were obtained from the MSigDB database (https://www.gsea-msigdb.org/gsea/msigdb) (8, 9). GSVA was used to assess the variation in pathway activity across different pancreatic islet cell types in T2D and ND samples. Using the GSVA R package, we performed a nonparametric, unsupervised analysis to calculate the enrichment scores for each hallmark gene set in individual cells. The enrichment scores represent the relative activity of specific pathways across various cell types, including macrophages, endocrine cells, and other cell populations within the islets. A higher score indicates greater pathway activity in the respective cell type. The results were visualized via heatmaps, which highlight the differential enrichment patterns in key pathways involved in inflammation, immune responses, and metabolism signaling in the T2D and ND samples. These cell type-specific pathway enrichments provide insights into the functional distinctions contributing to T2D pathogenesis.

### Machine learning for developing predictive models

We first identified differentially expressed genes (DEGs) by comparing macrophages in T2D and ND islet samples via the Wilcoxon rank-sum test. From the HP_ABNORMAL_BLOOD_MONOVALENT_INORGANIC_CATION_CONCENTRATION gene set, 149 abnormal blood monovalent inorganic cation concentration-related genes (ABRGs) were selected from the MSigDB database. By intersecting these ABRGs with the identified DEGs, we pinpointed the hub ABRGs for further analysis.

For the development of predictive models for T2D, we utilized two datasets: GSE54279 (22) and GSE41762 (23). GSE54279, generated from human samples via the GPL6244 platform, included 128 samples from T2D patients. GSE41762, also derived from human samples via the GPL6244 platform, comprises 77 samples, with 20 controls and 57 T2D patients. The microarray data from these datasets were preprocessed via the RMA method to correct for background, normalize the data, and adjust the probes. Batch effects were addressed via the Combat method.

To build predictive models for T2D, we evaluated 12 machine learning algorithms, creating 96 different model combinations. These algorithms included the LASSO, Ridge, Elastic network, Stepglm, SVM, GlmBoost, LDA, plsRglm, RSF, GBMs, XGBoost, and naive Bayes. The combinations were assessed via the AUC in both the training and validation cohorts. The models were constructed using the expression data of the hub ABRGs identified from the scRNA-seq analysis. Seventy percent of the samples from the combined GSE54279 and GSE41762 datasets were allocated for model training, whereas the remaining 30% were used for validation. The best-performing model was selected on the basis of its AUC score.

### Structural prediction of T2D-associated hub proteins via AlphaFold 3

To explore the structural characteristics of the hub proteins associated with T2D, we utilized AlphaFold 3 (24), a state-of-the-art tool for protein structure prediction. We selected a group of hub ABRGs linked to T2D, including *ATP1A1*, *CACNA1D*, *CALM1*, *CLDN10*, *NUP214*, *TDP2*, and *UNC93B1*, for detailed analysis. AlphaFold 3 was run with its default settings to ensure precise structural predictions. The primary amino acid sequences of the chosen proteins were input into AlphaFold 3, with multiple prediction iterations conducted for each protein to achieve reliable and consistent results. To evaluate the quality of the predicted structures, confidence scores such as pLDDT and pTM were calculated. A pTM score greater than 0.5 suggested a good structural match to the correct fold, whereas scores exceeding 0.8 indicated predictions of high confidence and accuracy.

### Correlation analysis between ABRGs

We examined the relationships between ABRGs via the “circlize” and “corrplot” R packages. To delve deeper into the associations between ABRGs and immune cell infiltration within T2D-affected islets, we applied Spearman correlation analysis. This approach allowed us to assess the correlation between the expression levels of ABRGs and the proportion of infiltrating immune cells.

### Immune cell infiltration and cytokine response analysis

Immune cell infiltration scores for the T2D and ND groups were calculated via single-sample gene set enrichment analysis (ssGSEA) with the “gsva” R package, which utilizes established immune cell markers (25). The infiltration patterns were visualized through heatmaps generated with the “pheatmap” package. Specifically, we focused on analyzing the relationships between 22 immune cell types and the expression levels of hub ABRGs (*ATP1A1*, *CACNA1D*, *CALM1*, *CLDN10*, *NUP214*, *TDP2*, and *UNC93B1*). Spearman’s correlation analysis was used to assess these relationships, with correlation coefficients (R) greater than 0.3 considered indicative of a strong positive correlation. The results were visualized via the “corrplot” and “ggplot2” R packages to provide a comprehensive view of the interactions between immune cell infiltration and hub gene expression in T2D and ND islet samples.

To further explore immune responses in macrophages, we employed a comprehensive cytokine response dictionary derived from data by Cui et al. (26), which captured transcriptional changes in response to stimulation by specific cytokines. We focused on 86 cytokines in islet samples from T2D and ND individuals and analyzed the enrichment scores (ESs) for each cytokine response via immune response enrichment analysis (IREA).

### Protein–protein interaction network construction

To elucidate the interactions among the 7 hub ABRGs, we constructed a PPI network using 50 genes closely associated with these hubs. The network analysis was performed via tools such as STRING (https://string-db.org/) (27) and Cytoscape (version 3.8.2), and significant interactions were visualized. A significance threshold was established with a confidence score exceeding 0.7. To further refine our understanding, six distinct algorithms—Closeness, Degree, EPC, MCC, MNC, and radiality—were applied to identify the top ten hub genes within the network. The UpSet plot was used to identify important proteins that were consistent across all six algorithms.

### Regulatory network analysis of hub genes

We explored the regulatory networks involving the 7 hub ABRGs and potential transcription factors (TFs) by integrating data from the JASPAR, ENCODE, and ChEA3 databases. The network was constructed and visualized via NetworkAnalyst (28), revealing complex interactions between the hub genes and TFs. A Venn diagram was used to show the overlap of the transcription factors across all three databases, indicating its central role in regulating these hub genes.

Finally, we conducted an analysis of the regulatory network between the 7 hub ABRGs and potential microRNAs (miRNAs) via the miRTarBase v9.0 and TarBase v9.0 databases. A Venn diagram illustrates the miRNAs shared between these two databases, suggesting their potential roles in modulating gene expression in T2D.

### Statistical analysis

All analyses were conducted via R (version 4.2.1). Statistical significance was defined as a P value less than 0.05.

## Results

### Overview of the study workflow

**Figure 1:**
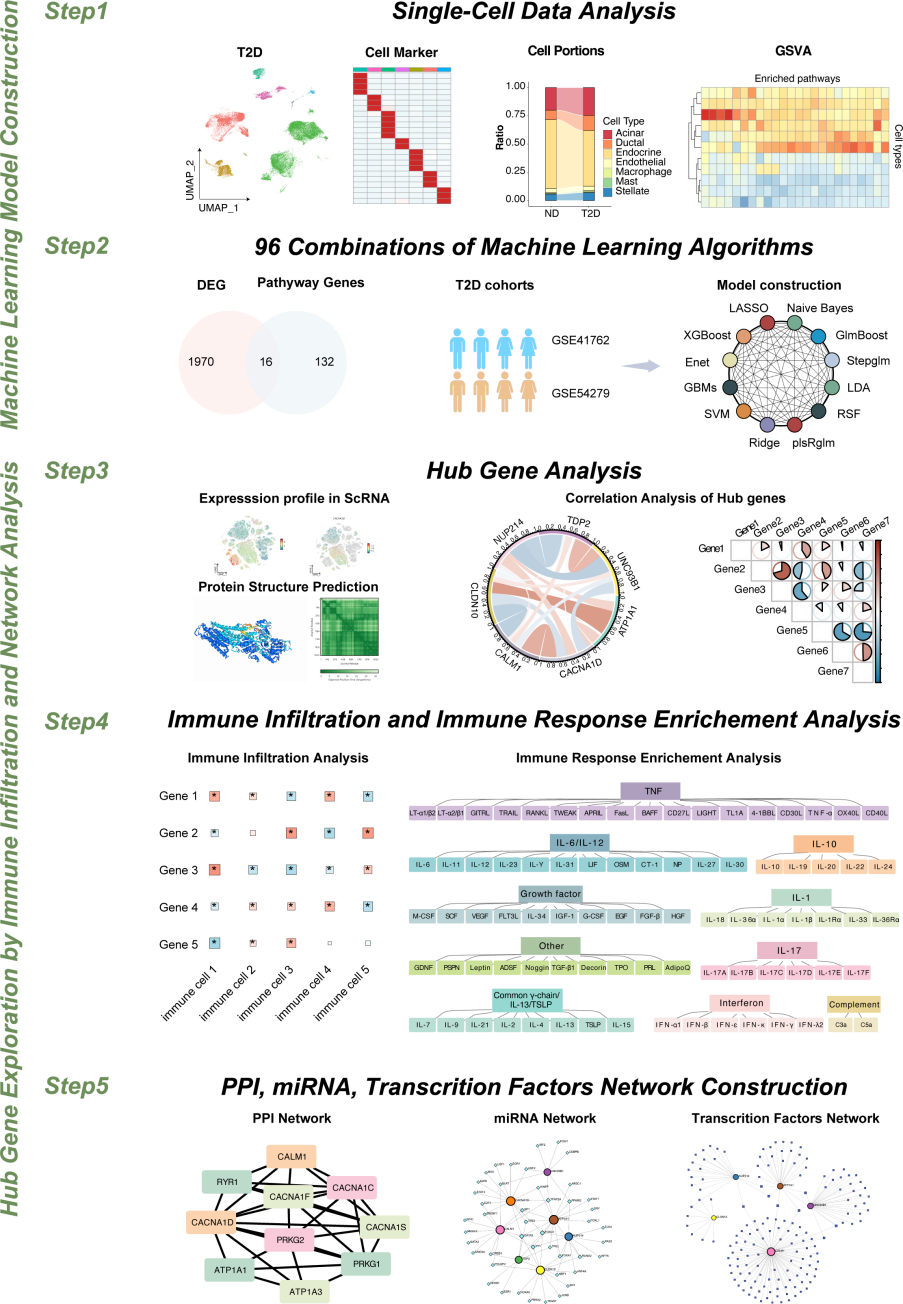
Research workflow overview.

This study was designed with a comprehensive workflow to explore the molecular mechanisms underlying type 2 diabetes (T2D) and its associated immune responses, particularly focusing on macrophages in pancreatic islet tissues (**Figure 1**).

Step 1: We began by performing single-cell RNA sequencing (scRNA-seq) on pancreatic islet cells from both T2D patients and nondiabetic (ND) individuals. These high-resolution data enabled us to identify differentially expressed genes (DEGs), which are key players in the immune landscape of islets, specifically in macrophages.

Step 2: We intersected these macrophage-specific DEGs with a predefined set of abnormal blood monovalent inorganic cation concentration-related genes (ABRGs) sourced from the MSigDB database. The overlap between the DEGs and ABRGs yielded a set of hub ABRGs, which were crucial for subsequent analyses. These hub ABRGs serve as the foundation for developing predictive models for T2D. We applied multiple machine learning algorithms to evaluate the performance of various models in distinguishing between T2D and ND samples. The best-performing model was selected on the basis of its accuracy, and this model was further utilized to deepen our understanding of the pathology of T2D.

To increase the functional relevance of these findings, we used AlphaFold 3 to predict the three-dimensional structures of the hub ABRG proteins. This structural information provides additional insights into the biological roles of these proteins in T2D.

Step 4: Further analysis was conducted to examine the relationships between these hub genes and immune cell infiltration, as well as cytokine responses, via single-sample gene set enrichment analysis (ssGSEA) and immune response enrichment analysis (IREA).

In step 5, we constructed protein–protein interaction (PPI) networks to elucidate the interactions among the hub genes, which provided insights into the molecular pathways involved in T2D. Finally, we expanded our investigation to include regulatory networks involving transcription factors (TFs) and microRNAs (miRNAs) that could modulate the expression of the identified hub genes. This comprehensive workflow, which combines scRNA-seq, DEG identification, ABRG intersection, machine learning, PPI network construction, and regulatory network analysis, provides a detailed landscape of the molecular mechanisms driving T2D, highlighting potential therapeutic targets for intervention.

### Gene expression profiling in pancreatic islet cells

**Figure 2:**
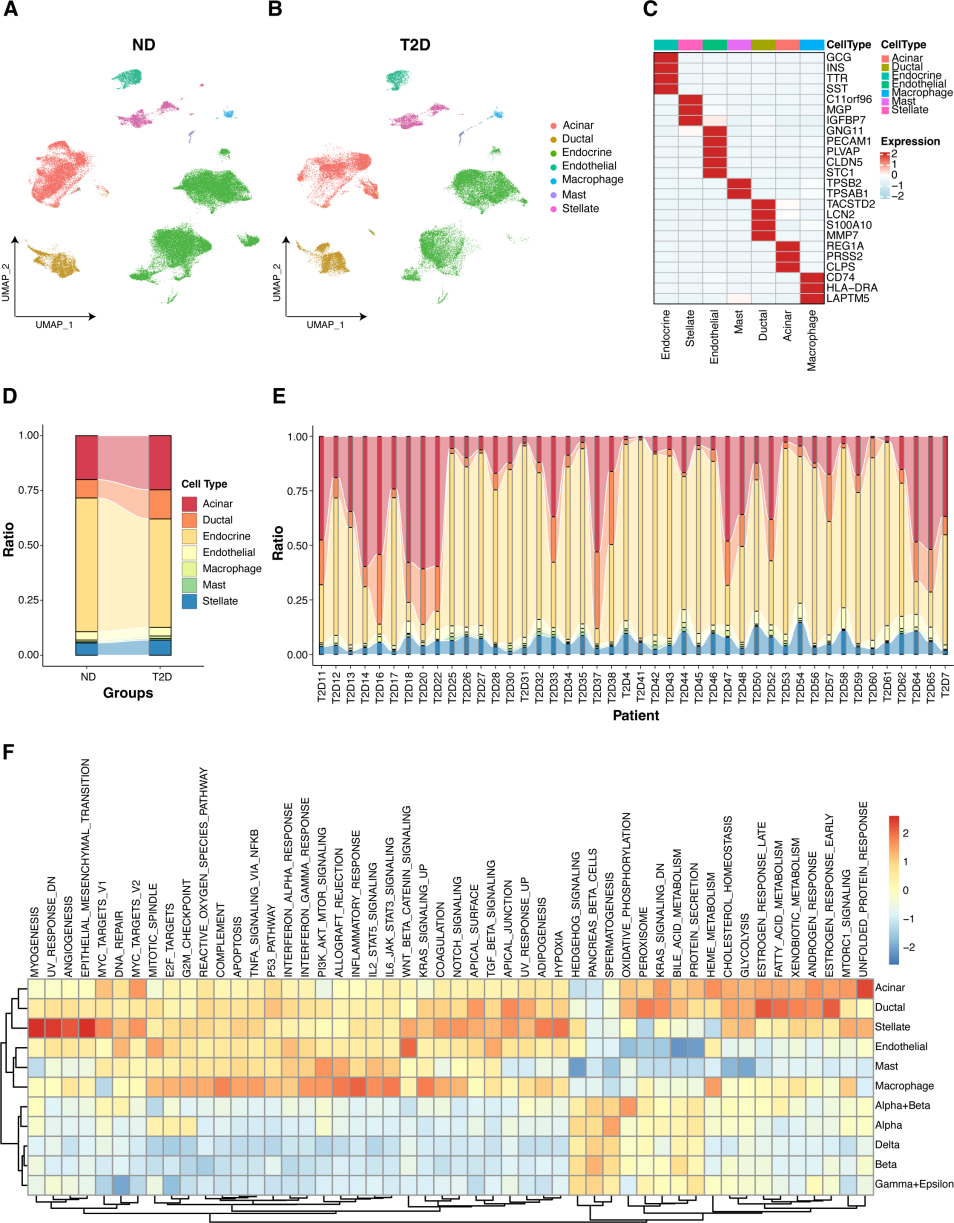
Gene expression visualization in pancreatic islet cells. **(A)** UMAP plot depicting the clustering of single-cell RNA-seq data from 27 nondiabetic (ND) individuals, highlighting the identification of seven primary islet cell types. **(B)** UMAP plot for single-cell RNA-seq data from 17 type 2 diabetes (T2D) patients, which also identified seven primary islet cell types. **(C)** Annotation of cell clusters on the basis of recognized marker genes, validating the identity of each cell type. **(D)** Comparative analysis of cell type distributions across T2D and ND samples categorized by disease state. **(E)** Individual-level comparison of cell type proportions in T2D and ND samples. **(F)** GSVA analysis illustrating the enrichment of hallmark gene sets across various cell types.

Using scRNA-seq data from 27 ND individuals and 17 T2D patients, we visualized gene expression across various pancreatic islet cell types. The uniform manifold approximation and projection (UMAP) plot revealed the clustering of cells into seven primary cell types, including endocrine cells, stellate cells, endothelial cells, mast cells, ductal cells, acinar cells, and macrophages, in both the ND and T2D samples (**Figures 2A, B**; **Supplementary Figure S1A**). Further analysis of these clusters revealed distinct subtypes within the primary populations (**Supplementary Figure S1B**). Annotation of these cell clusters, which was based on established marker genes, confirmed the identity of each cell type (**Figure 2C**). A comparative analysis of cell type proportions between T2D and ND samples highlighted significant differences, particularly in the distribution of endocrine cells, which are crucial for insulin secretion (**Figure 2D**). This analysis was further refined by evaluating the proportions at the individual level within each disease group, providing a detailed overview of the cellular heterogeneity in the islet samples (**Figure 2E**).

GSVA revealed the differential enrichment of hallmark gene sets across various cell types in both the T2D and ND samples (**Figure 2F**). Notably, key pathways associated with inflammation, metabolism, and the immune response presented distinct enrichment patterns in macrophages, endocrine cells, and other pancreatic islet cell types. Specifically, proinflammatory and metabolic pathways were more prominently enriched in macrophages from T2D samples, highlighting the role of macrophages in the inflammatory processes and metabolic dysregulation characteristic of T2D. Additionally, pathways related to insulin signaling and β-cell function were differentially enriched in endocrine cells, underscoring the functional impairment of insulin-producing cells in T2D. These findings provide valuable insights into the cell type-specific molecular mechanisms contributing to T2D pathogenesis.

### Development and validation of predictive models using machine learning

**Figure 3:**
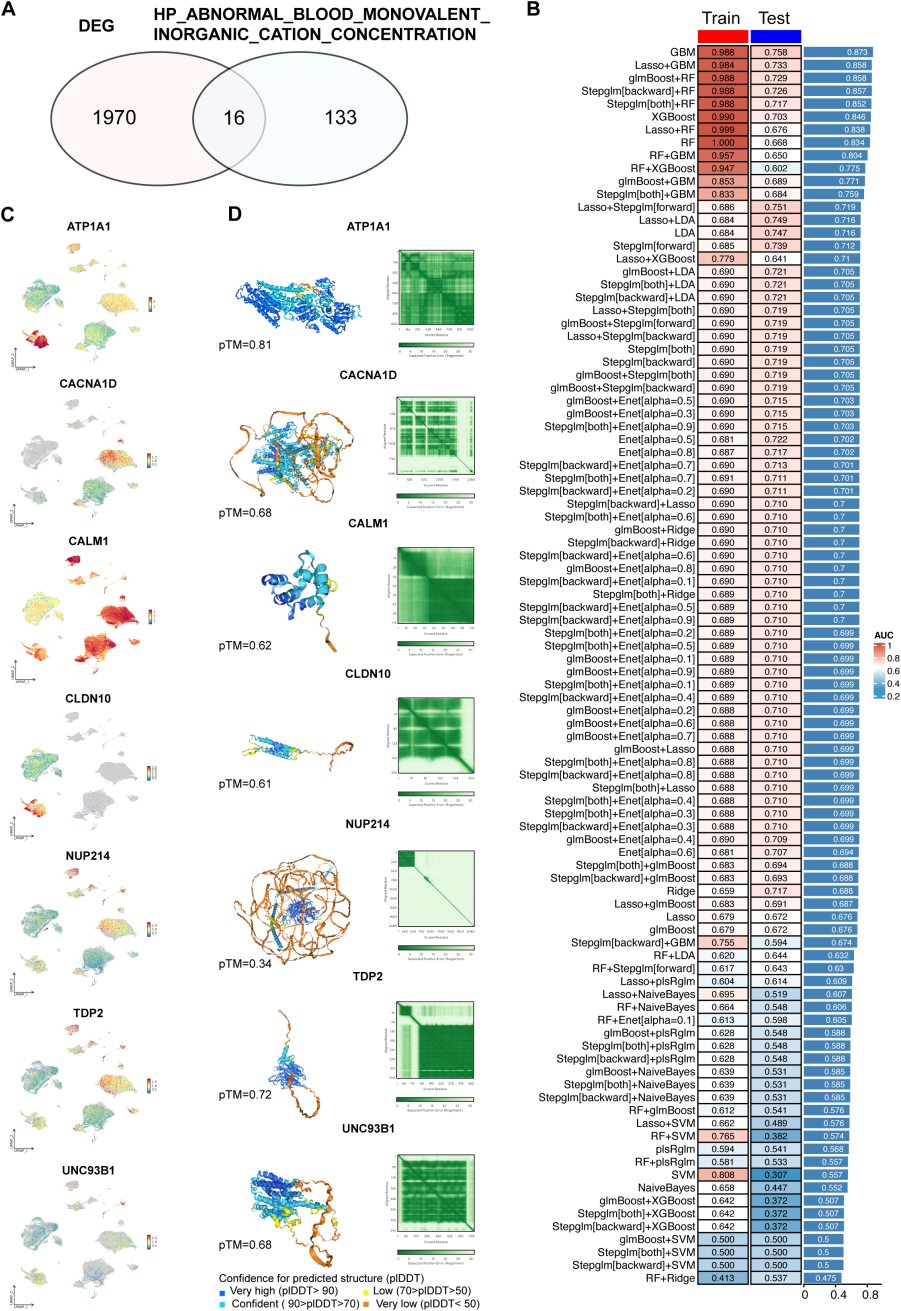
Development and validation of predictive models using machine learning. **(A)** Identification of 1986 differentially expressed genes (DEGs) between T2D and ND samples. From the HP_ABNORMAL_BLOOD_MONOVALENT_INORGANIC_CATION_CONCENTRATION gene set, 149 related genes were identified, with 16 intersecting with DEGs. **(B)** Performance metrics of 96 machine learning model combinations, where the GBM model demonstrated the highest accuracy in both the training (AUC=0.988) and validation (AUC=0.758) datasets. The average AUC for training and validation was 0.873. **(C)** UMAP plot displaying the expression patterns of hub genes related to abnormal blood monovalent inorganic cation concentrations. **(D)** Structural prediction of hub proteins via AlphaFold 3, with successful predictions for 7 hub proteins.

To develop predictive models for T2D, we first identified 1986 DEGs, specifically from macrophages in the pancreatic islet tissues of T2D and ND individuals (**Supplementary Table S1**). These DEGs were then cross-referenced with 149 genes associated with ABRGs (**Supplementary Table S2**), as identified from the MSigDB database. This intersection resulted in 16 overlapping ABRGs, which were selected as hub ABRGs for further analysis (**Figure 3A**, **Supplementary Table S3**).

Using these hub ABRGs, we tested a total of 96 machine learning model combinations to predict T2D. Among these models, the gradient boosting machine (GBM) model demonstrated superior performance, achieving an AUC of 0.988 in the training cohort and 0.758 in the validation cohort, with an average AUC of 0.873 across both cohorts (**Figure 3B**; **Supplementary Table S4**). To further explore the molecular profiles of these hub ABRGs, we applied UMAP visualization, which highlighted distinct expression patterns of the seven key hub genes identified by the GBM model (*ATP1A1*, *CACNA1D*, *CALM1*, *CLDN10*, *NUP214*, *TDP2*, *UNC93B1*) specifically in T2D samples (**Figure 3C**).

Additionally, structural predictions for these hub proteins were successfully generated via AlphaFold 3, providing valuable insights into their potential functional roles in T2D pathology (**Figure 3D**, **Supplementary Table S5**). ATP1A1 has a high confidence level (pTM = 0.81), indicating that it is a reliable target for downstream experimental analysis. Other proteins, such as TDP2 (pTM = 0.72), UNC93B1 (pTM = 0.68), CACNA1D (pTM = 0.68), CALM1 (pTM = 0.62), and CLDN10 (pTM = 0.61), also show moderate confidence, requiring further validation to ensure accuracy. Protein NUP214 (pTM = 0.34) has lower confidence levels, implying a need for cautious interpretation and more extensive validation. This approach, which combines DEG identification from macrophages, intersection with ABRGs, and machine learning, allowed us to construct and validate predictive models that provide a deeper insight of the mechanisms driving T2D.

### Hub gene correlations and immune characteristics in T2D

**Figure 4:**
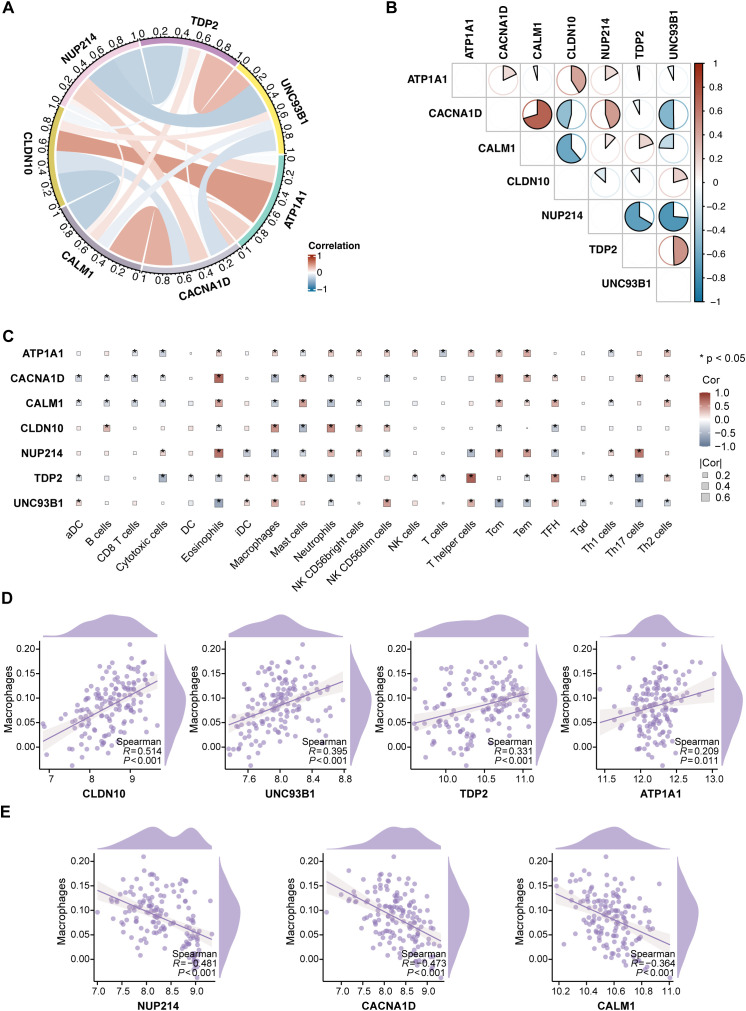
Characterization of immune features and hub genes in T2D. **(A)** Circular diagram depicting the relationships among the 7 hub genes, with red and blue lines indicating positive and negative correlations, respectively. **(B)** Correlation coefficients between the 7 hub genes, visualized through a pie chart. **(C)** Correlation analysis between the 7 hub genes and 22 immune cell types via ssGSEA, with significance levels marked as *P < 0.05. **(D)** Positive correlations between *CLDN10*, *UNC93B1*, *TDP2*, and *ATP1A1* expression and macrophage infiltration. **(E)** Negative correlations between *NUP214*, *CACNA1D*, and *CALM1* expression and macrophage infiltration.

We performed a comprehensive analysis of the functions of the 7 hub ABRGs in T2D. A circular diagram illustrating the relationships among these hub genes revealed both positive and negative correlations, with red and blue lines indicating these interactions, respectively (**Figure 4A**). For example, *UNC93B1* and *NUP214* demonstrated an antagonistic relationship (correlation coefficient, cor = -0.74), whereas *CACNA1D* and *CALM1* exhibited a significant synergistic interaction (cor = 0.70) (**Figure 4B**; **Supplementary Table S6**).

Further correlation analysis was performed to explore the associations between the hub ABRGs and 22 immune cell types in T2D patients via ssGSEA (**Figure 4C**; **Supplementary Table S7**). Notably, a positive correlation was observed between macrophage infiltration and the expression of *CLDN10*, *UNC93B1*, *TDP2*, and *ATP1A1* (**Figure 4D**). In contrast, *NUP214*, *CACNA1D*, and *CALM1* were negatively correlated with macrophage infiltration (**Figure 4E**). These results highlight the intricate interplay between immune responses and gene expression in T2D, providing insights into the potential roles these hub genes play in modulating immune characteristics within the diabetic environment.

### Cytokine signature enrichment in macrophages

**Figure 5:**
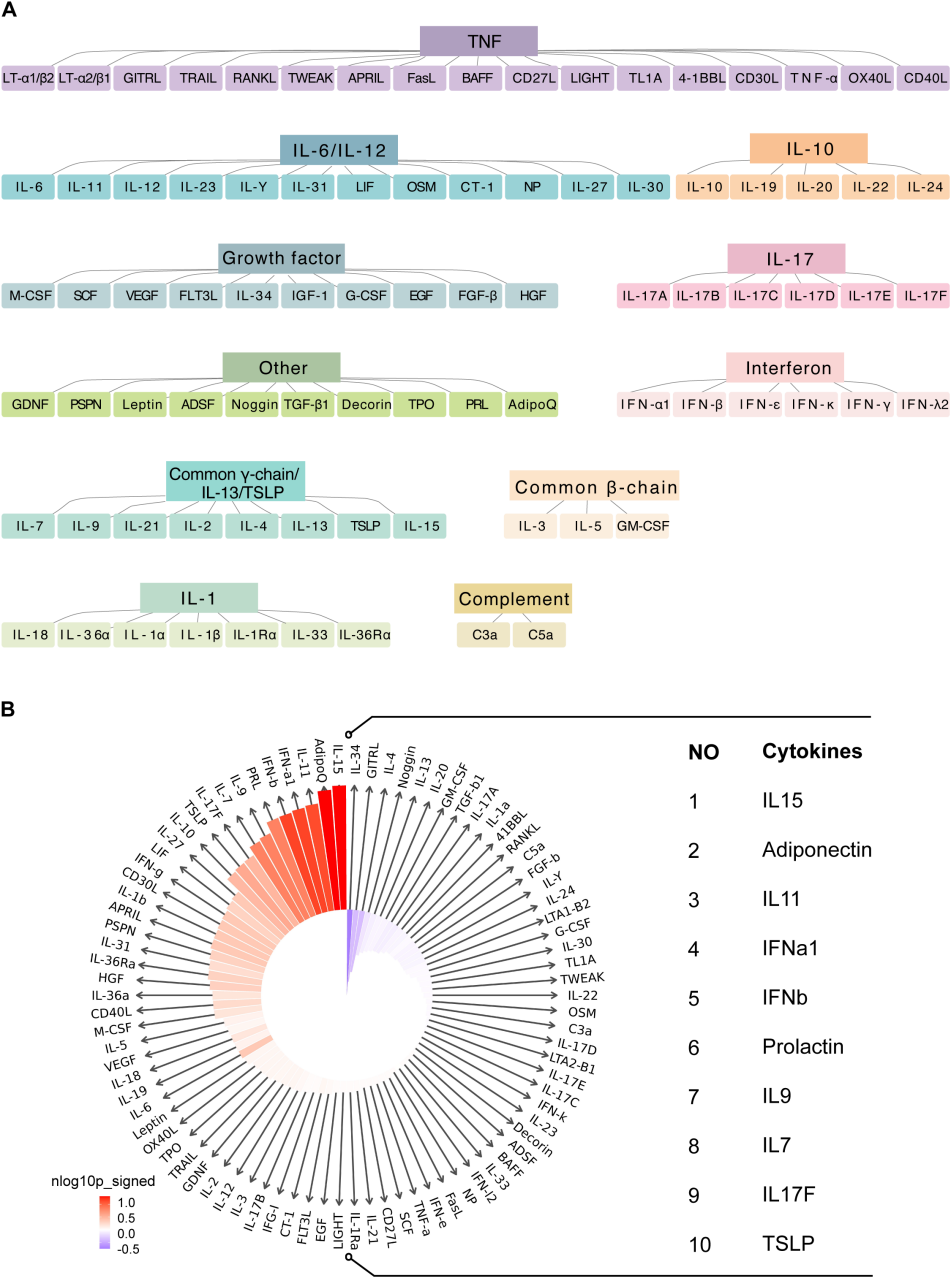
Cytokine signature enrichment in macrophages. **(A)** Utilizing a comprehensive cytokine response dictionary. **(B)** The IREA cytokine enrichment plot presents the enrichment score (ES) for each of the 86 cytokine responses in macrophages from T2D versus ND islet samples.

To explore immune responses in macrophages, we utilized cytokine response dictionary, derived from the dataset compiled by Cui et al. (26). We focused on the responses of islet samples from T2D and ND individuals to 86 cytokines (**Figure 5A**). The IREA cytokine enrichment plot displayed enrichment scores (ESs) for each cytokine response in macrophages, comparing T2D versus ND islet samples. The results revealed significant cytokine signatures in T2D, with the shading bars indicating the false discovery rate (FDR)-adjusted P values.

For macrophages, we observed dominant enrichment in several key cytokines, including IL15, adiponectin, IL11, IFNα1, IFNβ, prolactin, IL9, IL7, IL17F, and TSLP (**Figure 5B**; **Supplementary Table S8**). These cytokines are known to play critical roles in modulating immune responses and inflammation: IL15 is involved in the activation and proliferation of NK cells and T cells, potentially contributing to the elevated immune activation observed in T2D. Adiponectin is a hormone with anti-inflammatory properties; however, its role in macrophages in the context of T2D might reflect a compensatory response to inflammation. IL11 is typically associated with fibrotic responses, which may be related to tissue remodeling and fibrosis in diabetic islets. Type I interferons (IFNα1 and IFNβ) are key players in antiviral responses, but their enrichment in T2D suggests a chronic inflammatory state that could exacerbate autoimmunity or metabolic dysfunction. Prolactin has been implicated in immune regulation, and its involvement here could reflect its role in modulating inflammatory pathways in T2D. IL9 and IL7 are cytokines that support the survival and function of various immune cells, potentially contributing to the sustained immune activity observed in T2D islets. IL17F is part of the IL-17 cytokine family, which is known for promoting inflammatory responses; its enrichment could be linked to the proinflammatory environment in T2D. Thymic stromal lymphopoietin (TSLP) is involved in the activation of dendritic cells and T-helper cells, possibly driving immune responses in the diabetic pancreas.

This enrichment of cytokine responses in macrophages underscores their significant role in the inflammatory microenvironment of T2D. The identified cytokines likely contribute to the chronic inflammation characteristic of T2D, which can impair insulin signaling and β-cell function, further exacerbating the disease.

### PPI network of the hub genes

**Figure 6:**
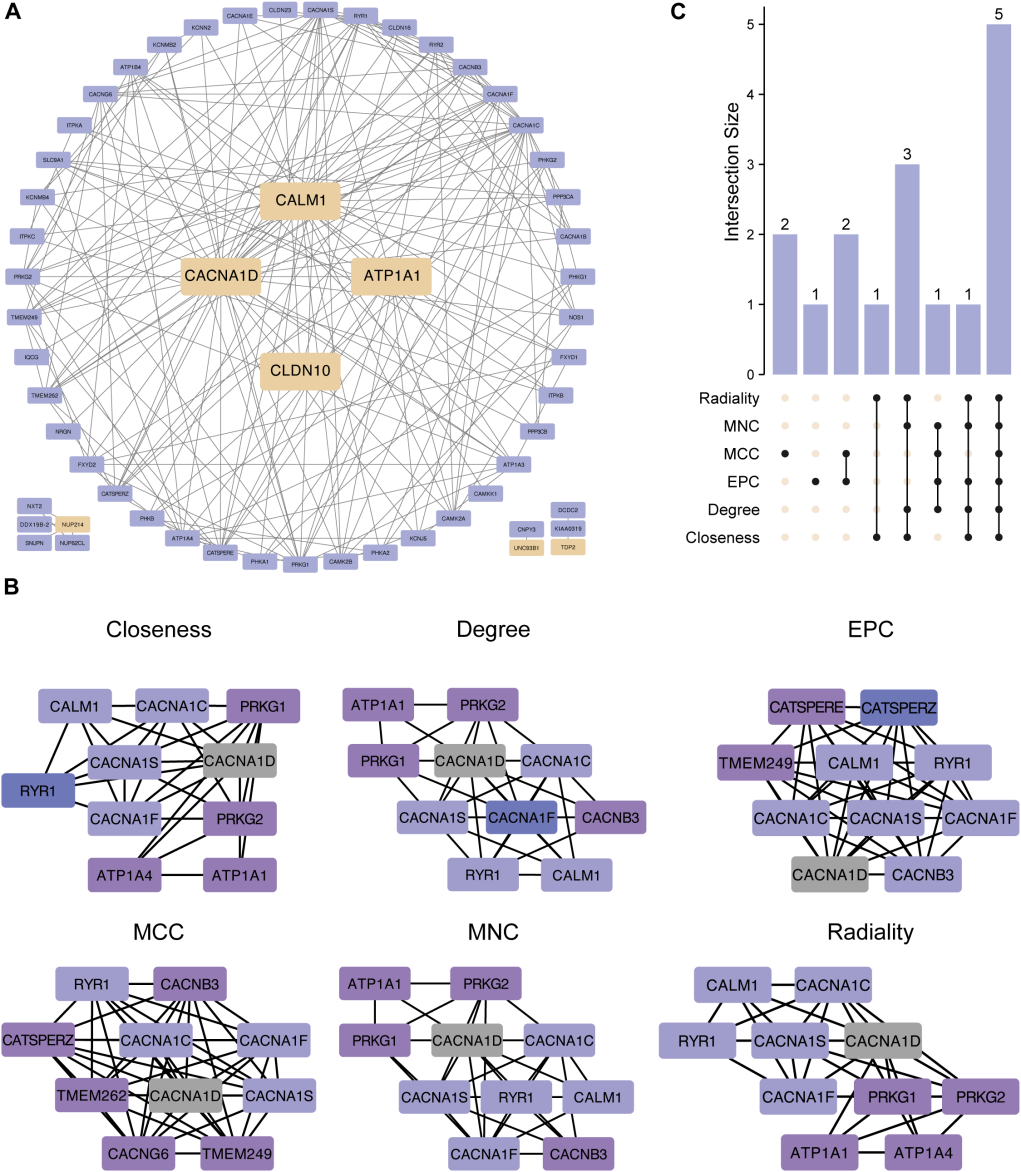
Protein–protein interaction (PPI) network of the hub genes. **(A)** PPI network created from 50 genes closely linked to the 7 hub genes, showing gene interactions. CALM1, CACNA10, ATP1A1, and CLDN10 were notably interconnected, whereas NUP214, UNC93B1, and TDP2 exhibited more independent behavior. **(B)** The top ten hub genes were identified via six algorithms: closeness, degree, EPC, MCC, MNC, and radiality. **(C)** UpSet plot showing 5 shared proteins in all six algorithms.

We constructed a PPI network using 50 genes closely linked to the 7 hub ABRGs, which revealed significant interactions among these genes (**Figure 6A**; **Supplementary Table S9**). Notably, CALM1, CACNA10, ATP1A1, and CLDN10 were highly interconnected, suggesting their collaborative role in T2D pathogenesis (**Figure 6A**). In contrast, NUP214, UNC93B1, and TDP2 appeared to function more independently (**Figure 6A**). To further refine our understanding of these interactions, we used six distinct algorithms—Closeness, Degree, EPC, MCC, MNC, and Radiality —to identify the top ten hub genes, which could serve as potential targets for therapeutic intervention (**Figure 6B**; **Supplementary Table S10**). The UpSet plot revealed 5 shared proteins (CACNA1D, CACNA1C, CACNA1S, CACNA1F, and RYR1) among all six algorithms (**Figure 6C**, **Supplementary Table S11**).

### Regulatory network of hub genes and TFs

**Figure 7:**
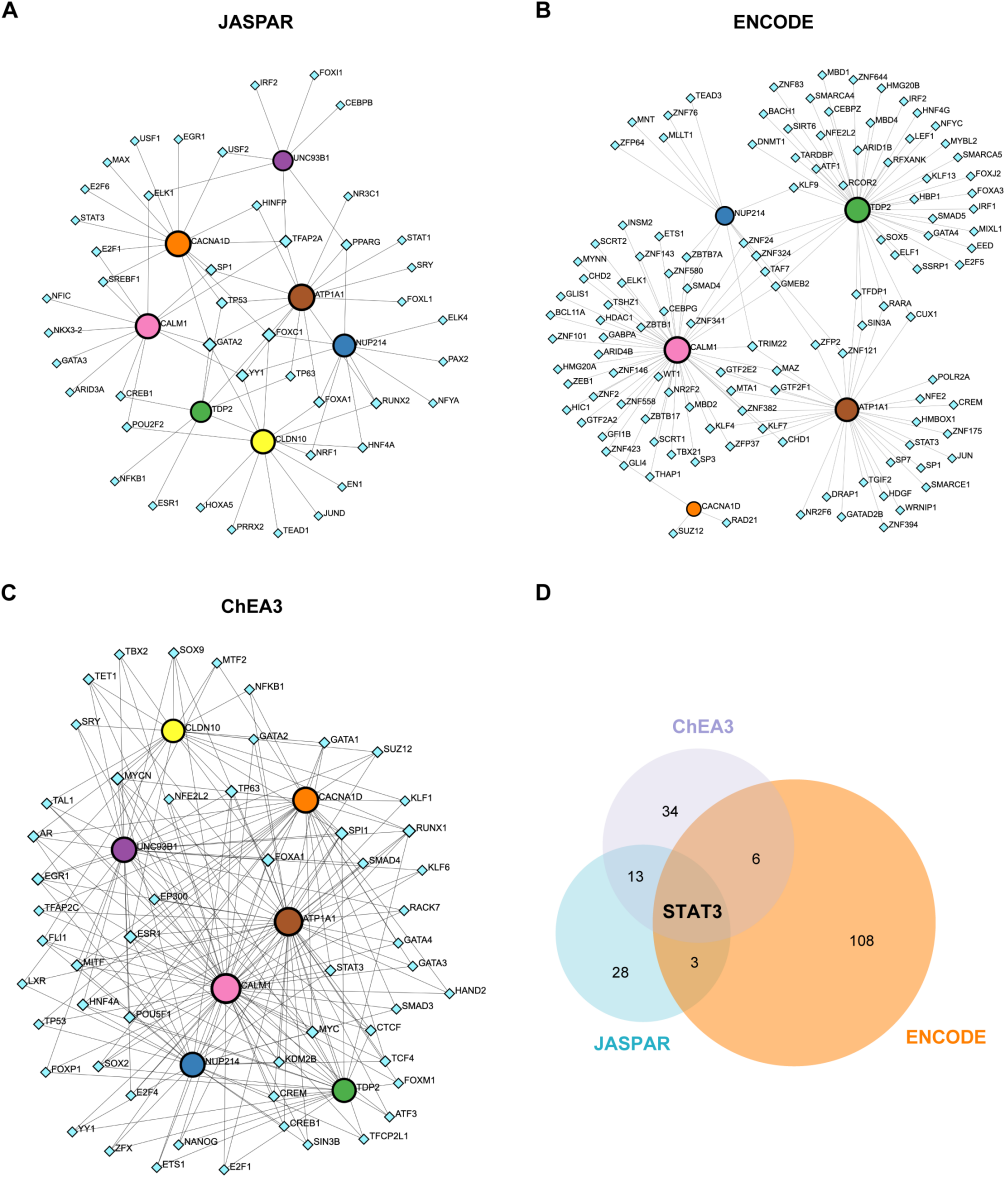
Regulatory network of hub genes and transcription factors (TFs). **(A)** Circular nodes represent hub genes, and diamond-shaped nodes represent TFs. Interactions between the 7 hub genes and potential TFs were mapped via the JASPAR database, revealing 52 nodes and 79 edges. **(B)** Network analysis based on the ENCODE database, showing 123 nodes and 144 edges. **(C)** Network analysis using the ChEA3 database, identifying 61 nodes and 213 edges (degree filter=2.0). **(D)** Venn diagram illustrating the overlap of the TF STAT3 across the JASPAR, ENCODE, and ChEA3 databases.

We explored the regulatory network between the 7 hub ABRGs and potential TFs via data from the JASPAR, ENCODE, and ChEA3 databases. The network analysis identified a complex web of interactions, with circular nodes representing hub ABRGs and diamond-shaped nodes representing TFs. A total of 52 nodes and 79 edges were identified in the JASPAR database (**Figure 7A**; **Supplementary Table S12**), 123 nodes and 144 edges in the ENCODE database (**Figure 7B**; **Supplementary Table S12**), and 61 nodes and 213 edges in the ChEA3 database (degree filter=2.0, **Figure 7C**; **Supplementary Table S12**). A Venn diagram further revealed the overlap of STAT3 across all three databases, indicating its central role in regulating these hub genes (**Figure 7D**; **Supplementary Table S13**).

### Regulatory network of hub genes and miRNAs

**Figure 8:**
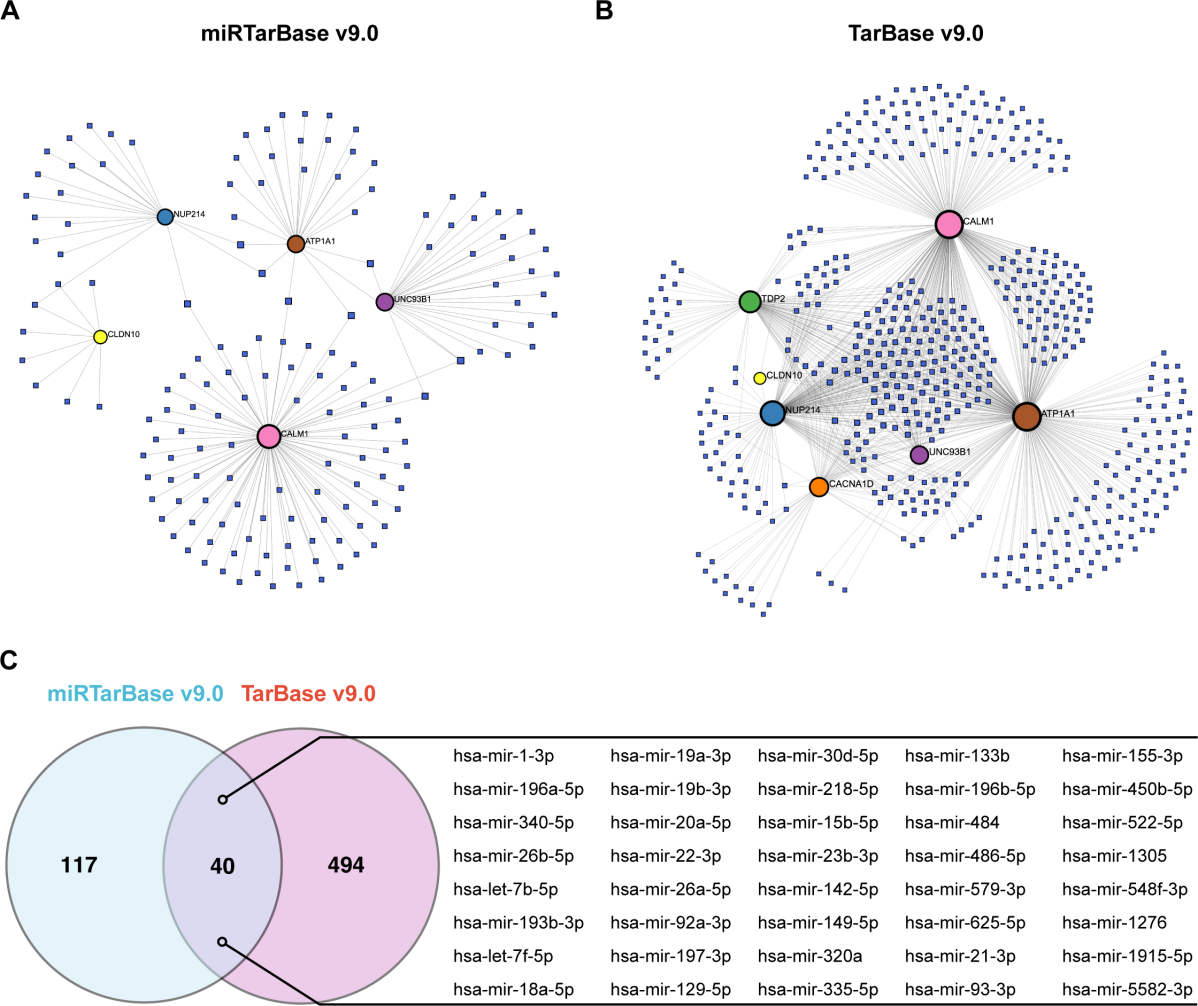
Regulatory network of hub genes and miRNAs. **(A)** Circular nodes represent hub genes, and square nodes represent miRNAs. Network analysis based on the miRTarBase v9.0 database identified 52 nodes and 79 edges. **(B)** Analysis via the TarBase v9.0 database revealed 541 nodes and 1082 edges. **(C)** Venn diagram showing the 40 miRNAs shared between the miRTarBase v9.0 and TarBase v9.0 databases.

Finally, we analyzed the regulatory network between the 7 hub ABRGs and potential miRNAs via the miRTarBase v9.0 and TarBase v9.0 databases. The network analysis identified 52 nodes and 79 edges in the miRTarBase v9.0 database (**Figure 8A**; **Supplementary Table S14**) and 541 nodes and 1082 edges in the TarBase v9.0 database (**Figure 8B**; **Supplementary Table S14**). A Venn diagram illustrated the 40 miRNAs, such as hsa-mir-1-3p, hsa-mir-196a-5p, and hsa-mir-340-5p, that were shared between these two databases, suggesting their potential regulatory roles in modulating gene expression in T2D (**Figure 8C**; **Supplementary Table S15**).

## Discussion

In this study, we employed a comprehensive approach to elucidate the molecular mechanisms underlying type 2 diabetes (T2D), with a particular focus on the role of macrophages in pancreatic islet tissues. By integrating single-cell RNA sequencing (scRNA-seq), machine learning, and network analysis, we aimed to identify key genes associated with T2D, explore their functional relevance, and uncover potential therapeutic targets.

Our investigation began by leveraging scRNA-seq data to identify differentially expressed genes (DEGs) in macrophages from T2D and nondiabetic (ND) pancreatic islet tissues. Macrophages are known to play a critical role in the inflammatory processes that characterize T2D. The immune response enrichment analysis (IREA) in our study revealed that macrophages in T2D samples presented a distinct cytokine signature, notably including enrichment of proinflammatory cytokines such as IL15, IFNα1, IFNβ, and IL17F. These cytokines are closely associated with the proinflammatory M1 phenotype of macrophages (29–31). The observed cytokine enrichment suggests that macrophages in T2D are more inclined to adopt a proinflammatory state, which has been linked to β-cell dysfunction and insulin resistance. This finding aligns with previous studies (32, 33), providing robust evidence for the proinflammatory shift of macrophages in T2D. These DEGs formed the basis for further analysis aimed at revealing the molecular underpinnings of T2D.

A key innovation in our study was the intersection of macrophage-specific DEGs with abnormal blood monovalent inorganic cation concentration-related genes (ABRGs). ABRGs have been increasingly recognized for their role in various metabolic and inflammatory pathways related to T2D pathophysiology. By focusing on these genes, we identified 16 overlapping hub ABRGs that are likely central to the disease process. These hub genes not only reflect the dysregulation of ion homeostasis in T2D but also underscore the importance of macrophages in mediating these effects.

To further explore the significance of these hub ABRGs, we developed predictive models for T2D via machine learning techniques. Among the 96 model combinations tested, the gradient boosting machine (GBM) model emerged as the most effective, demonstrating high accuracy in distinguishing between T2D and ND samples. The hub genes identified by GBM, including *ATP1A1*, *CACNA1D*, *CALM1*, *CLDN10*, *NUP214*, *TDP2*, and *UNC93B1*, underscore the potential of machine learning in identifying robust biomarkers for T2D and the utility of integrating omics data with advanced computational methods.

Among the hub genes identified, several have established links to T2D through their roles in pancreatic beta-cells and systemic metabolic regulation. For instance, ATP1A1 (encoding the α1 subunit of the Na+/K+-ATPase) and CACNA1D (encoding the α1 subunit of the Cav1.3 L-type calcium channel) are known to influence ion homeostasis and have been implicated in insulin secretion and β-cell function (34, 35). Additionally, CALM1 (calmodulin 1), a key mediator of calcium signaling, has been linked to various metabolic processes, including glucose metabolism (36). However, our study specifically identified ATP1A1, CACNA1D, and CALM1 as DEGs in macrophages from T2D pancreatic islets when compared to non-diabetic individuals, and as hub ABRGs. This shifts the focus towards their potential involvement in macrophage functions within the T2D microenvironment.

Our investigation into their roles in macrophages revealed several key findings. Firstly, the altered expression of ATP1A1, CACNA1D, and CALM1 in macrophages from T2D patients suggests these ion homeostasis-related genes are also pertinent to macrophage biology or are indicative of macrophage dysfunction in T2D. Secondly, we explored their correlation with macrophage infiltration. Our analysis showed a positive correlation between the expression of ATP1A1 and macrophage infiltration in T2D, suggesting that higher ATP1A1 expression in macrophages might be associated with an increased presence or a particular activity state of macrophages contributing to the inflammatory environment characteristic of T2D. Conversely, we observed that CACNA1D and CALM1 expression levels were negatively correlated with macrophage infiltration in T2D patients. This intriguing finding could imply several possibilities: these genes might be downregulated in specific pro-inflammatory macrophage populations prevalent in T2D islets; their decreased expression could be part of a disrupted regulatory mechanism within macrophages in the T2D microenvironment; or it may reflect a shift in macrophage subpopulations, where those with higher expression of CACNA1D and CALM1 are less abundant.

The identification of these genes as DEGs in macrophages, coupled with their established roles in ion homeostasis, implies they are part of the molecular machinery within these immune cells that could influence inflammatory responses or other macrophage functions relevant to T2D. ABRGs, as a group, are involved in various physiological processes relevant to T2D, including inflammatory signaling pathways. Therefore, our study highlights that ATP1A1, CACNA1D, and CALM1, being dysregulated in macrophages, likely contribute to T2D pathogenesis through their involvement in the immune response and inflammation mediated by these cells. While our current findings primarily point to the association of these genes with macrophage states and infiltration in T2D, further research is warranted to elucidate the precise molecular mechanisms by which ATP1A1, CACNA1D, and CALM1 modulate macrophage phenotype and function (e.g., cytokine production, phagocytosis, or antigen presentation) in the context of type 2 diabetes. Our findings lay the groundwork for such future investigations by highlighting these genes as relevant players in macrophages in T2D.

On the other hand, several hub genes identified in our study—namely CLDN10 (claudin 10), NUP214 (nucleoporin 214), TDP2 (tyrosyl-DNA phosphodiesterase 2), and UNC93B1 (unc-93 homolog B1)—have not been previously extensively associated with T2D. Our investigation provides new insights into their potential roles, particularly within macrophages in the T2D context. All four of these genes were identified as DEGs in macrophages from T2D pancreatic islets in our study, forming a primary basis for discussing their involvement in the disease.

Our findings suggest that these genes may play novel roles in T2D pathogenesis, particularly through their involvement in the immune response and inflammation mediated by macrophages. Specifically, our correlation analysis revealed distinct associations with macrophage infiltration: CLDN10 expression showed a positive correlation with macrophage infiltration in T2D. This finding, coupled with literature connecting CLDN10 with immune infiltration in other contexts, supports its potential relevance to macrophage activity and the inflammatory environment in T2D. UNC93B1 expression also demonstrated a positive correlation with macrophage infiltration. UNC93B1 is recognized as an innate-immune related gene, further substantiating its potential role in macrophage biology and immune responses within the diabetic islets. TDP2 expression was similarly positively correlated with macrophage infiltration in T2D. While specific prior literature on TDP2’s role in macrophages is not extensively cited in this study beyond its identification as a DEG, its positive correlation with macrophage presence suggests it may contribute to the inflammatory microenvironment mediated by macrophages in T2D. In contrast, NUP214 expression was found to be negatively correlated with macrophage infiltration in our T2D samples. This novel finding suggests a distinct role for NUP214 concerning macrophage presence or phenotype in T2D, possibly being downregulated in certain macrophage populations or involved in a disrupted regulatory pathway.

Collectively, the differential expression of these genes in macrophages and their varied correlations with macrophage infiltration highlight them as novel players in macrophage pathobiology in T2D. Their dysregulation likely contributes to T2D pathogenesis via macrophage-mediated immune and inflammatory processes. While for CLDN10 and UNC93B1, existing literature supports their immune functions (37, 38), the precise roles and mechanisms of all four genes, particularly TDP2 and NUP214, within macrophages in the T2D setting warrant more specific investigation. Their identification and initial characterization in this study provide a strong foundation for such future research.

We also utilized AlphaFold 3 for structural prediction of the hub proteins, which provided additional insights into their functional relevance. The predicted three-dimensional structures of these proteins offer a basis for future studies aimed at understanding their specific roles in T2D and designing targeted therapies. Notably, structural analysis revealed potential interaction sites with other proteins, suggesting that these hub ABRGs could be involved in complex molecular networks driving T2D pathology.

Our exploration of protein–protein interaction (PPI) networks further supported this notion. PPI analysis revealed significant interactions among the hub genes, particularly those involving CALM1, CACNA1D, ATP1A1, and CLDN10, which were highly interconnected. These findings suggest that these genes may work collaboratively to regulate key pathways involved in T2D. In contrast, other hub genes, such as NUP214, UNC93B1, and TDP2, appeared to function more independently, indicating that multiple distinct pathways contribute to the disease.

The correlation analysis between hub ABRGs and immune cell infiltration revealed important insights into the interplay between gene expression and immune responses in T2D. Specifically, several hub genes, including *CLDN10* (37), *UNC93B1* (38), and *ATP1A1* (39), were positively correlated with macrophage infiltration. These findings suggest that these genes may contribute to the inflammatory environment in T2D, further exacerbating the disease. The cytokine enrichment analysis provided additional evidence for this, showing that macrophages in T2D exhibit a distinct cytokine signature that likely drives the chronic inflammation characteristic of the disease.

Our regulatory network analysis involving transcription factors (TFs) and microRNAs (miRNAs) highlighted the complex regulatory mechanisms governing the expression of hub ABRGs in T2D. Among the TFs identified, STAT3, known for its multifaceted roles in inflammatory signaling and β-cell function, has been extensively reported in relation to T2D (40). STAT3 is crucial for maintaining β-cell homeostasis by modulating the cell cycle and protecting against DNA damage, as evidenced in models of pancreatic injury (41). Additionally, STAT3 plays a vital role in β-cell survival by negatively regulating the PTEN-AKT signaling pathway, which is critical for preventing β-cell apoptosis under hyperglycemic conditions (42). Moreover, STAT3 has been implicated in mediating β-cell epithelial–mesenchymal transition in the context of chronic pancreatitis-related diabetes, where its activation promotes β-cell dedifferentiation and loss, further contributing to diabetes progression (43). These findings collectively underscore the importance of STAT3 in both the maintenance of β-cell function and the pathogenesis of T2D.

Similarly, hsa-mir-1-3p (44) and hsa-mir-340-5p (45) are miRNAs that have been previously implicated in metabolic regulation and T2D pathogenesis, suggesting their involvement in modulating the expression of genes critical to the disease. In addition to these known regulators, our study identified several novel miRNAs that have not been previously associated with T2D. For example, hsa-mir-196a-5p is among the newly identified miRNAs that may play roles in the regulatory networks driving T2D, potentially influencing macrophage activity and β-cell function. The discovery of these novel regulatory molecules provides novel insights into the transcriptional and posttranscriptional control of T2D-associated genes. Understanding these regulatory networks could open new avenues for therapeutic intervention by targeting specific TFs or miRNAs to modulate gene expression in T2D. This approach may lead to more precise strategies for controlling inflammation, preserving β-cell function, and ultimately managing or preventing the progression of T2D.

However, this study has several limitations that should be acknowledged. The scRNA-seq analysis was based on a relatively small clinical cohort (27 ND and 17 T2D individuals), a common challenge in studies involving human pancreatic islets, which may impact the broader generalizability of the initial DEG findings. The observed drop in AUC for our machine learning model from the training (0.988) to the validation set (0.758), while still indicating reasonable predictive performance of the hub ABRGs, suggests some overfitting and underscores the need for model validation and refinement in larger, more diverse cohorts. Moreover, our conclusions on the roles of these hub genes in T2D are largely based on correlative and computational analyses. While these methods are powerful for hypothesis generation, future studies employing genetic association data from resources like the UK Biobank and utilizing Mendelian Randomization approaches would be invaluable to help establish causal relationships between these hub genes and T2D risk.

Furthermore, the primary objective of this study was to employ a comprehensive bioinformatics approach, integrating scRNA-seq data analysis, machine learning, and network biology, to uncover novel genes and potential therapeutic pathways. Consequently, the identification of the seven hub ABRGs (ATP1A1, CACNA1D, CALM1, CLDN10, NUP214, TDP2, and UNC93B1) and the construction of their intricate regulatory networks involving transcription factors and miRNAs were completed based on analyses of existing databases and computational methods. We acknowledge that these predictions and network constructions, while providing valuable insights and generating strong hypotheses, warrant further investigation through experimental validation to substantiate their functional roles and therapeutic potential. For instance, as noted, while structural predictions for ATP1A1 showed high confidence, other proteins require further validation.

Crucially, the specific regulatory roles of the identified hub genes in macrophage functions—such as their direct impact on ion channel activity or cytokine secretion—and the confirmation of their expression levels and patterns *in vivo*, require direct experimental confirmation. While our study proposes these hub genes as potential novel therapeutic targets, their detailed clinical implications, including potential interactions with existing T2D therapies such as SGLT2 inhibitors, remain to be elucidated. Future translational research will need to explore these aspects once the functional roles and relevance of these targets are more firmly established.

While conducting extensive new wet-laboratory experiments (e.g., using *in vitro* cell models, animal models of T2D, or patient-derived samples) is beyond the scope of the current manuscript, which focuses on the initial discovery and nomination of these targets through advanced computational analysis, we firmly believe that our study provides a robust and detailed foundation for such future experimental work. The identified hub genes and regulatory pathways are now high-priority candidates for these targeted validation studies. Similarly, while our study proposes these hub genes as potential novel therapeutic targets, their detailed clinical implications, including potential interactions with existing T2D therapies such as SGLT2 inhibitors, remain to be elucidated and represent an important avenue for future translational research once their functional roles are more firmly established.

Understanding these regulatory networks and the functions of the identified hub genes could reveal opportunities for therapeutic intervention by targeting specific genes, TFs, or miRNAs to modulate gene expression in T2D. This approach may lead to more precise strategies for controlling inflammation, preserving β-cell function, and ultimately managing or preventing the progression of T2D.

In conclusion, our study provides a detailed landscape of the molecular mechanisms driving T2D, emphasizing the central roles of macrophages and ABRGs in the disease process. The integration of scRNA-seq, machine learning, and network analysis has enabled us to identify key genes and pathways contributing to T2D and to propose potential therapeutic targets. The findings presented here offer a strong foundation for future experimental validation and translational research aimed at developing novel therapeutic strategies for T2D.

## Data Availability

The original contributions presented in the study are included in the article/**Supplementary Material**. Further inquiries can be directed to the corresponding authors.

## Supplementary material

Supplementary Figure 1 UMAP visualization of pancreatic islet cell clusters. **(A)** UMAP plot of singlecell RNA-seq data from 27 nondiabetic (ND) and 17 type 2 diabetes (T2D) individuals, identifying seven primary islet cell types. **(B)** UMAP plot of the same dataset, highlighting distinct subtypes within the islet cell populations.
