# Supplementary figures and images for: Targeting macrophages and ion homeostasis in T2D: new genes and therapeutic pathways identified
Source: Front Immunol. 2025 Aug 14;16:1514243. doi: 10.3389/fimmu.2025.1514243 (PMC12391075; doi:10.3389/fimmu.2025.1514243)

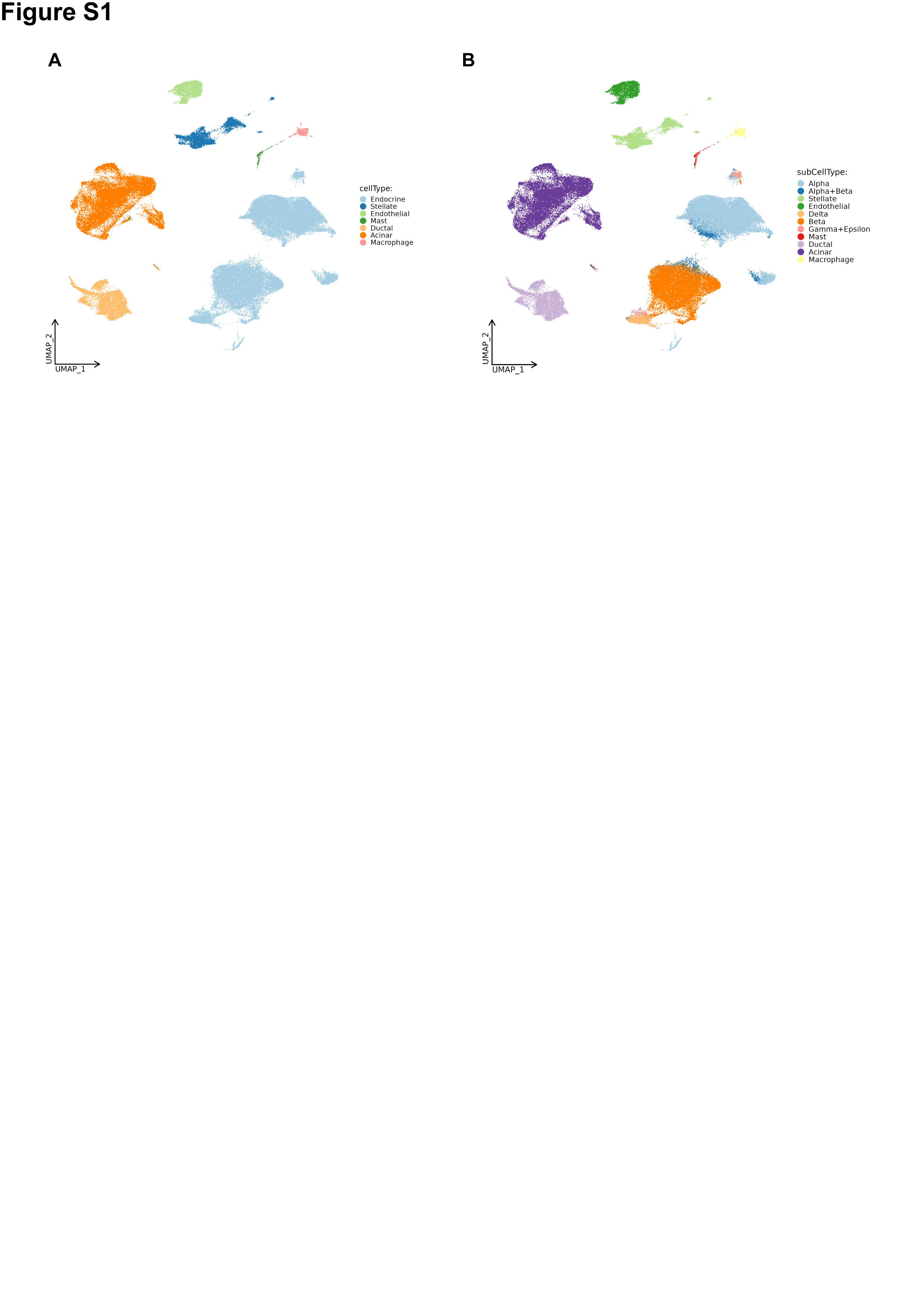

Supplement: Supplementary Figure 1 — UMAP visualization of pancreatic islet cell clusters. (A) UMAP plot of singlecell RNA-seq data from 27 nondiabetic (ND) and 17 type 2 diabetes (T2D) individuals, identifying seven primary islet cell types. (B) UMAP plot of the same dataset, highlighting distinct subtypes within the islet cell populations. [file Image1.tif]
